# Implementation of Chest Compression Feedback Technology to Improve the Quality of Cardiopulmonary Resuscitation in the Emergency Department: A Quality Initiative Test-of-change Study

**DOI:** 10.7759/cureus.5523

**Published:** 2019-08-29

**Authors:** Jodie Pritchard, Jillian Roberge, Joseph Bacani, Michelle Welsford, Shawn Mondoux

**Affiliations:** 1 Emergency Medicine, McMaster University, Hamilton, CAN; 2 Emergency Medicine, McMaster University Hospital, Hamilton, CAN

**Keywords:** cardiopulmonary resuscitation, emergency treatment, feedback, quality improvement

## Abstract

Background

Cardiopulmonary resuscitation (CPR) metrics including compression rate and depth are associated with improved outcomes and the need for high-quality CPR is emphasized in both the American Heart Association (AHA) and Heart and Stroke Foundation of Canada (HSFC) guidelines. While these metrics can be utilized to assess the quality of CPR, they are infrequently measured in an objective fashion in the emergency department.

Objectives

As part of an Emergency Department (ED) Quality Improvement (QI) project, we sought to determine the impact of real-time audio-visual (AV) feedback during CPR amongst ED healthcare providers.

Methods

Participants performed two minutes of uninterrupted CPR without AV feedback, followed by two minutes of CPR with AV feedback after a two-minute rest period in a simulated CPR setting. CPR metrics were captured by the defibrillator and uploaded to review software for analysis of each event.

Results

The use of real-time AV feedback resulted in a significant improvement in the number of participants meeting AHA/HSFC recommended depth (38%, p = 0.0003) and rate (35%, p = 0.0002). Importantly, ‘compressions in target’, where participants met both rate and depth simultaneously, improved with AV feedback (19 vs 61%, p < 0.0001).

Conclusions

We found a significant improvement in compliance with CPR depth and rate targets as well as ‘compressions in target’ with the use of real-time AV feedback during simulation training. Future research is needed to ascertain whether these results would be replicated in other settings. Our findings do provide a robust argument for the implementation of real-time AV CPR feedback in Hamilton Emergency Departments.

## Introduction

Cardiopulmonary resuscitation (CPR) metrics including compression rate and depth are associated with improved outcomes and can be utilized to assess the quality of CPR administered [1-4]. The majority of research surrounding CPR feedback effectiveness is performed in the out-of-hospital cardiac arrest (OHCA) environment. Survival-to-discharge rates in OHCA are reduced by 30% when depth of chest compression is <38 mm [1]. In-hospital research shows that return of spontaneous circulation (ROSC) rates fall from 72% to 42% when CPR is delivered too slowly [2]. Similarly, a systematic review and meta-analysis of both in- and out-of-hospital cardiac arrest found that deeper chest compressions and rates closer to 100 were significantly associated with improved survival rates [3].

While the American Heart Association (AHA) and Heart & Stroke Foundation of Canada (HSFC) guidelines emphasize the need for high-quality CPR, this is infrequently measured in an objective fashion in the emergency department (ED) [5]. Studies looking at the subjective visual assessment of chest compression quality, performed during simulated resuscitation scenarios, demonstrated that practitioners could not reliably identify the correct performance of target compression rates, nor adequate depth of compression, though AV-feedback improved depth perception [6,7]. A hospital-based study of CPR performed on mannequins, found that only 22% of chest compressions met AHA guidelines for both rate and depth [8]. Given the importance of high-quality CPR, and the evidence that feedback improves compressions by healthcare staff, several defibrillator manufacturers have introduced technological devices which provide real-time feedback [9]. A 2013 study using real-time audio-visual (AV) feedback in OHCA showed that survival increased by 5.5% to 13.9% overall, after real-time feedback introduction, with an adjusted OR 2.78 (95% CI 1.15-6.41) [4]. As part of an ED Quality Improvement (QI) project, we sought to determine the impact of providing real-time feedback during CPR amongst experienced ED healthcare providers.

## Materials and methods

Real-time CPR AV feedback deployment was planned for the ED at Hamilton Health Sciences (HHS), a tertiary care academic facility comprised of two adult and one pediatric hospitals in Ontario, Canada. The Juravinski site sees approximately 45,000 primarily adult ED patients per year, of which approximately 65 are adult patients in cardiac arrest. The ED utilizes Zoll R series defibrillators that are capable of providing real-time CPR feedback, however at the time of our study it did not stock the supplies required for this function and so the first step was to employ the CPR AV feedback during simulation training.

Participants were recruited from the Juravinski ED’s annual interprofessional training for nursing and other staff. All subjects included had previous CPR training and were >18 years of age. Exclusion criteria involved any participant that had previous training with real-time CPR AV feedback. A total of 45 individuals were enrolled. The majority were nurses whose job regularly entails the provision of CPR within the ED.

Each participant was asked to perform two minutes of uninterrupted CPR on a mannequin without access to the AV feedback prompts from the defibrillator, to simulate their current practice in the ED. This was immediately followed by a two-minute period of rest, then an additional two minutes of compressions with the real-time AV feedback enabled. No feedback was given to the participants by the investigators on the quality of their initial or feedback-augmented CPR performance. Participants were given a brief introduction, prior to the start of the study, on the defibrillator AV feedback function. The sequence of CPR without AV feedback followed by with AV feedback was standardized for all participants to minimize the learned effect of CPR performance.

The data from each two-minute session of CPR for each participant was recorded by the defibrillator and stored, with no participant identifying information, on an internal memory card. This data was then analyzed using Zoll’s proprietary RescueNet Code Review program (Zoll Medical Corporation, 2018, version 5.8.1). This software allowed interpretation of the discreet two-minute CPR sessions, looking specifically at the rate, depth, and ‘compressions in target’, which refers to a compression that has both the correct rate and depth (overall quality for each compression) as per AHA/HSFC 2015 guidelines and is reported as a percentage of total compressions during a period of time. Recoil feedback is available as a "release" bar on the Zoll defibrillator display, however no audio prompts are given unlike those with rate and depth of compressions. Our study participants had no previous exposure to real-time AV feedback prompting and so we chose to limit our initial analysis to rate and depth of compressions, for which the defibrillator provided both visual and audible feedback prompts. Feedback CPR was performed on the Life/form® Deluxe “Plus” CRiSis™ Manikin with the Zoll CPR stat-padz multifunction defibrillation pads with built-in accelerometer.

RescueNet Code Review data was exported to Excel (Microsoft Excel for Mac, Office 365 subscription, 2019, version 16.26) for further analysis. Compressions in target were analyzed using a paired t test. Depth and rate averages for each two-minute CPR cycle were analyzed for standard deviation as well as the proportion change to meet AHA/HSFC defined correct values. AHA/HSFC 2015 guidelines recommend a compression rate of 100-120 and depth of 5-6 cm (2.0-2.4 inches) [5]. MedCalc software (www.medcalc.org) was used to calculate statistical values.

## Results

The results of the CPR quality with and without real-time AV feedback are presented in Table 1. Of the 45 participants in our study, only 18 (40%) were able to provide CPR of correct depth without AV feedback, but this increased to 35 (78%) with the addition of AV feedback. Similarly, the compliance with compression rates during CPR improved from 25 (56%) without AV feedback to 41 (91%) with feedback.

**Table 1:** CPR Metrics with and without Feedback *Target compliance difference measures the difference seen in number of participants meeting correct average depth or rate. cpm: compressions per minute; CPR: Cardiopulmonary resuscitation.

	Without AV Feedback	With AV Feedback	Target Compliance Difference*	p-value
Depth average (cm)	5.31 (SD 0.46)	5.49 (SD 0.19)	38% improvement 95% CI (17.79-54.15)	P = 0.0003
Rate average (cpm)	115.33 (SD 13.13)	111.22 (SD 6.30)	35% improvement 95% CI (17.05-50.39)	P = 0.0002
Compressions in Target (%)	19	61		P < 0.0001

We assessed the target compliance difference, which measures the difference seen in the number of participants meeting the correct average rate or depth. This was utilized as both rate and depth are continuous variables within a selected acceptable range, making the use of their means for comparison ineffective. The use of real-time AV feedback resulted in significant improvement in the number of participants meeting the AHA/HSFC recommended depth (p = 0.0003) and rate (p = 0.0002). We also found an improvement in ‘compressions in target’ from 19% without feedback to 61% with AV feedback (p < 0.0001) amongst our study participants thus demonstrating a statistically significant improvement with the use of real-time AV feedback.

## Discussion

Quality CPR improves survival. The most effective CPR occurs when both the correct depth and rate are simultaneously achieved, along with a chest compression fraction of >60%. Our results demonstrated that in simulated practice, ED staff rarely meet overall CPR guidelines without AV feedback, but frequently meet target quality metrics when using AV feedback. These statistically significant improvements demonstrate that among this population of experienced health care providers and with minimal training, the percentage of chest compressions fully compliant with AHA/HSFC guidelines can be dramatically improved by the implementation of real-time AV feedback.

Our study specifically focuses on ED provider CPR quality, an area for further research. A previous study utilizing AV-feedback as part of a bundle of initiatives to improve CPR performance found improved CPR quality and adherence to guidelines in an academic ED [10]. The use of real-time AV feedback in our study reduced the variability seen in both the rate and depth of compressions, with fewer CPR events falling outside of the guidelines and by smaller amounts, as evidenced by the significant improvement in compliance difference for both rate and depth and tighter standard deviations. Previous studies have found a significant narrowing of CPR variable distributions when utilizing real-time AV feedback [11].

We looked at the rate, depth, and ‘compressions in target’, which refers to a compression that has both the correct rate and depth (overall quality for each compression) and is reported as a percentage of total compressions over a period of time. While no literature has demonstrated that adequate ‘compressions in target’ is associated with improved survival, studies have utilized similar combination of variables as a marker for CPR quality. Several studies have used a measure of ‘efficient chest compressions’ as defined by the composite of adequate frequency, depth, and release force in a simulated environment [12,13]. These studies have shown improvement in ‘efficient chest compressions’ with the use of real-time AV feedback amongst professional and non-CPR trained participants, though patient outcomes were not measured. A study by Peberdy et al. examined the performance of CPR in a simulated setting in-hospital, measuring rate, depth, and the percentage of chest compressions meeting both targets. Similar to our study, they found that a much lower percentage of chest compressions met both rate and depth guidelines [8].

A 2014 study showed that chest compression fraction (CCF) >60% was associated with an odds ratio (OR) for survival-to-discharge three times higher than those receiving CCF <20% [14]. Included in the 2016 study analyzing observer assessment of rate and depth, findings illustrated that observers are unable to assess adequate CCF unaided [6]. Evidence shows that in the OHCA, mean CCF increased from 66.2% to 83.7% with the use of real-time AV feedback and we anticipate similar improvements in the hospital environment [4]. Our study design did not assess CCF given the two-minute CPR analysis design, however, we anticipate that during the full course of a cardiopulmonary resuscitation it would have a similar beneficial effect as seen in the OHCA environment. Future studies of CPR quality either in simulation or in situ should assess this to determine if real-time AV feedback in the ED setting can improve CCF.

Our study measured the provision of CPR in a controlled, single task, simulated setting. Given the complexities of providing cardiac arrest care, including CPR, in the ED environment, it is likely that the quality of CPR delivered without feedback is less than was seen in our study. It is conceivable that a larger effect may be measured in the actual ED environment with multiple distractions, emotions, and competing interests.

Limitations

Our CPR quality data was obtained in a simulated, unblinded setting which may have introduced the observer effect. Hawthorne bias, where the participants may perform better knowing they are being observed, cannot be accounted for in our study design. We anticipate that this effect would also have occurred in the control group and thus is unlikely to negate our finding of significant improvement in CPR quality with real-time AV feedback.

A complete assessment of the value of this technology would require in vivo testing. In this simulated setting, it was possible to identify individual CPR providers and compare their individual performance with and without real-time AV feedback which would not have been possible in an actual resuscitation.

CPR was performed for two minutes without AV feedback, followed by two minutes rest and then two minutes of CPR with AV feedback for each participant. We allowed participants two minutes of rest between attempts in order to minimize fatigue on CPR performance in their second evaluation. It is possible that performance of the initial two minutes of CPR without AV feedback improved the performance of the subsequent CPR, although, since no feedback was given, we do not anticipate this to be a significant effect. Previous studies have demonstrated no learning effect when performing CPR without AV-feedback, while significant learning effect was seen when performing CPR with AV-feedback [8]. A randomized, cross-over study of Paramedic students demonstrated improvement in CPR quality with audio-feedback that was sustained in the cross-over to no-feedback arm; similar effects were not seen in the control group without feedback [15]. Similarly, another study demonstrated improvement in compression depth with AV feedback training that was retained at three months [16]. Future studies, if performed in a similar simulated setting, could randomize participants to CPR with or without AV feedback first.

Limitations to generalizability of our study include the small number of participants, simulated setting, and the single center design. Simulation lacks the emotion and stressors of a live resuscitation. Regardless of setting we anticipate the data to overestimate the quality of CPR in the control group and thus underestimate the improvement in compliance during a live resuscitation. We anticipate effects such as fatigue to affect both control and AV-feedback groups, and although AV-feedback is likely to detect decreasing compliance more effectively, it is possible that the additional data from the AV-feedback could increase resuscitation complexity decreasing its ability to improve performance. Although the number of participants and single site design impacts the generalizability of our findings beyond our site, the significant improvement we observed in ‘compressions in target’ suggests that the provision of real-time AV feedback was able to improve the quality of CPR provision (19 vs 61%, p < 0.0001) in our setting and justifies a broader implementation study.

## Conclusions

We found a significant improvement in compliance with CPR depth and rate targets as well as ‘compressions in target’ with the use of real-time AV feedback during ED simulation training. This study is a first step in demonstrating the role for this tool in the ED setting. Future research is needed to ascertain whether these results would be replicated in other settings. Our findings do provide a robust argument for the implementation of real-time AV CPR feedback in Hamilton Emergency Departments.
